# Using Audience Response Technology to provide formative feedback on pharmacology performance for non-medical prescribing students - a preliminary evaluation

**DOI:** 10.1186/1472-6920-12-113

**Published:** 2012-11-13

**Authors:** Alison Mostyn, Oonagh Meade, Joanne S Lymn

**Affiliations:** 1 School of Veterinary Medicine and Science, University of Nottingham, Sutton Bonington Campus, Loughborough, UK; 2 School of Nursing, Midwifery & Physiotherapy, University of Nottingham, Queens Medical Centre, Nottingham, UK

**Keywords:** Audience response technology, Teaching, Pharmacology, Non-medical prescribing

## Abstract

**Background:**

The use of anonymous audience response technology (ART) to actively engage students in classroom learning has been evaluated positively across multiple settings. To date, however, there has been no empirical evaluation of the use of individualised ART handsets and formative feedback of ART scores. The present study investigates student perceptions of such a system and the relationship between formative feedback results and exam performance.

**Methods:**

Four successive cohorts of Non-Medical Prescribing students (n=107) had access to the individualised ART system and three of these groups (n=72) completed a questionnaire about their perceptions of using ART. Semi-structured interviews were carried out with a purposive sample of seven students who achieved a range of scores on the formative feedback. Using data from all four cohorts of students, the relationship between mean ART scores and summative pharmacology exam score was examined using a non-parametric correlation.

**Results:**

Questionnaire and interview data suggested that the use of ART enhanced the classroom environment, motivated students and promoted learning. Questionnaire data demonstrated that students found the formative feedback helpful for identifying their learning needs (95.6%), guiding their independent study (86.8%), and as a revision tool (88.3%). Interviewees particularly valued the objectivity of the individualised feedback which helped them to self-manage their learning. Interviewees’ initial anxiety about revealing their level of pharmacology knowledge to the lecturer and to themselves reduced over time as students focused on the learning benefits associated with the feedback.

A significant positive correlation was found between students’ formative feedback scores and their summative pharmacology exam scores (Spearman’s rho = 0.71, N=107, p<.01).

**Conclusions:**

Despite initial anxiety about the use of individualised ART units, students rated the helpfulness of the individualised handsets and personalised formative feedback highly. The significant correlation between ART response scores and student exam scores suggests that formative feedback can provide students with a useful reference point in terms of their level of exam-readiness.

## Background

Audience response technology (ART), often referred to as personal response units or ‘clickers’, has been demonstrated to have a positive impact on teaching and learning in a number of educational areas, including clinical subjects such as pharmacy, medicine, veterinary medicine, physiotherapy and nursing [1-8]. As reviewed by Jones et al., the use of ART promotes an active approach to learning, provides prompt formative feedback, increases interaction and opportunities for reflection on knowledge and is accessible to students with diverse learning styles [9]. All of these aspects increase information retention and promote ‘deeper’ and active approaches to learning [1,5,7,10,11]. Indeed, recent literature has reported that ART is easy to use [4], promotes participation and attention in class [2,12] and increases confidence levels [3,6,13].

Promoting knowledge and confidence in pharmacological knowledge in non-medical prescribing (NMP) students is crucial to developing effective and safe prescribing professionals. In the U.K., upon successful completion of a six month non-medical prescribing (NMP) course, certain health professionals, including nurses, have similar independent prescribing rights to doctors [14]. Pharmacology is the largest component of the NMP course accounting for more than a third of the taught hours. However, this is a subject with which nurses in particular have struggled [15-17].

Our recent data have suggested that students attending the NMP course found the use of the “KeePad” ART in pharmacology lectures beneficial both in terms of promoting understanding of key concepts as well as integration of concepts [8]. The most prevalent theme within student feedback on the use of the ART was that the system was helpful in terms of identifying learning needs [8]. A number of studies have demonstrated that students value being able to respond to questions anonymously through these systems [3,5,18]. Whilst the anonymous use of ART allows students to immediately relate their performance to that of the rest of the cohort, students do not receive longer-term detailed individual feedback when the system is used in this anonymous manner.

The distribution of personal ART handsets to students is not uncommon but as far as the authors are aware, these personal handsets have only been used to monitor lecture attendance rather than to provide individual feedback [11]. Data from a study on medical students’ perceptions of ART suggests that the system should only be used in the anonymous format in order to reduce student anxiety in relation to using this technology [3]. In terms of using formative assessment to improve student performance, however, the provision of objective feedback on an individual’s performance is likely to be key [19,20]. It has been suggested that the most effective type of feedback for students is that which is both detailed and individual [reviewed in [21]. Indeed, the introduction of frequent objective feedback from progress tests resulted in a reduction in failure rates for medical students at McMaster University [20]. Although histograms of class results can be made available electronically during or following a lecture using the anonymous ART system [11], this does not provide students with an objective measure of their own performance over the long term. Students are unlikely to be able to recall accurately their performance on each ART session and they are unlikely to be able to remember which answers they provided to each question.

The current study is the first that the authors are aware of to utilise an ART system in a personal rather than anonymous format and to provide detailed, objective, individual feedback to students throughout the duration of the NMP course. The aims of this study are to both explore student perceptions of the use of personal ART handsets and the associated individual formative feedback, and to determine whether there is a relationship between ART scores and student summative assessment scores. By examining the relationship between ART scores and exam results, we can examine how helpful ART feedback may be in guiding students towards understanding their level of exam readiness.

## Methods

### Participants

All students (n= 107) from four successive cohorts (September 2009 – September 2011) of the Non-Medical Prescribing course at the University of Nottingham had access to individual ART units for the purposes of this study. Questionnaire data was collected from three cohorts of students (n=72) and seven students were invited for interview.

### Use of ART in pharmacology lectures

ART units were distributed to students at the beginning of each pharmacology lecture and collected at the end. Each ART was matched with an individual student, so that each individual used the same handset in all pharmacology sessions to answer exam-style questions as previously described [8]. Immediately after students had answered the questions using their ART unit, the correct answer was displayed, along with a summary of the number of responses made by the class.

**Figure 1:**
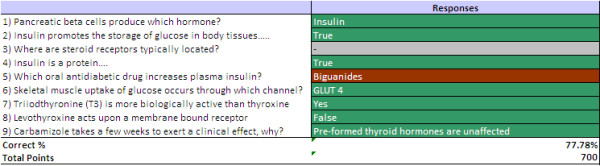
**Example of individualised feedback sheet.** A green box indicates a correct response from the student; red, an incorrect response and grey, an unanswered question. In this session, the student achieved 78% successful answers.

The use of individual handsets allowed the lecturers to track and record each student’s progress over the entire pharmacology course. Lecturers sent weekly e-mails to students containing feedback about their performance on the ART questions. This feedback enabled students to view the questions asked during the lecture, and to see whether they had answered each question correctly or incorrectly. Correct answers to questions were also supplied. An example feedback sheet is included in Figure 1.

### Questionnaire

Student perceptions of using the individualised ART units were initially assessed using a questionnaire distributed to all students (n=79) in three successive cohorts (September 2009, January 2010, September 2010) at the end of the course. The questionnaire contained demographic questions and fixed response questions relating to students’ perceptions of using the individualised ART units and the individual feedback. The questionnaire used was identical to the one described in our previous study on anonymous ART use within a NMP course [8], with the addition of a section which asked students about whether or not they accessed the individual feedback they received, whether they used this feedback and their perceptions of the usefulness of this feedback. Students were asked to rate their level of agreement with a number of statements regarding the usefulness of the individual feedback for promoting their learning, identifying their learning needs, directing their independent study and as a revision tool.

### Interviews

Students from a single cohort were invited to take part in semi-structured interviews about their experiences of using the individualised ART. The sampling strategy was purposive in order to select students who scored differently on their individual ART feedback and may therefore have had different experiences of the ART system due to their potentially different learning needs. A group of students comprising two who had consistently scored over 80% across all lecture weeks, three who consistently scored between 70-79% each week and two who consistently scored less than 70% each week were recruited. These categories were based on the pass mark of the pharmacology exam which is set at 80%.

Interviews lasted between 20–30 minutes and were conducted by a research assistant who was not affiliated with the students’ teaching team. Interview questions centred on the following: students’ initial reactions to the ART, their experience of using the response units in the classroom, their use/non-use of the formative feedback which was made available to them and the perceived advantages and disadvantages of this technology and the associated feedback for their learning. Interviews were analysed using inductive thematic analysis.

### Statistical analysis

An overall mean score for each student on the ART questions was calculated from each student’s feedback sheets. Students’ mean scores on the ART system were correlated with their summative pharmacology exam scores using a two-tailed Spearman’s rho calculation.

### Ethical approval

This study was approved by the Medical School Ethics Committee at the University of Nottingham (Project Code H/05/2010).

## Results

### Questionnaire

72 out of 79 students completed the questionnaire on student perceptions of the individualised ART system (response rate of 91%). The respondents were mainly female (81%), and in the 31–40 (47%) or 41–50 (40%) age bracket. The majority of students rated their pre-course biological knowledge as either “poor” (40%) or “moderate” (47%), with a minority of students rating their prior biological knowledge as “good” (10%) or “excellent” (3%).

In terms of student experience of using the ART in the classroom, 97% of participants answered that they enjoyed using the system. Students’ levels of agreement with statements about the perceived usefulness of using ART in the classroom are presented in Table 1.The majority of students agreed with statements regarding the usefulness of ART for: identifying learning needs (95.9%); maintaining focus in the lecture (93.1%); stimulating their interest in the lecture (90.3%) and allowing the lecturer to check student understanding (95.8%). Fewer students felt that the use of this system motivated them to revise before the next lecture (66.7%). While 97.2% of students agreed that the use of ART helped them to gain an idea of exam-style questions, less than half of the students (43.1%) felt that the use of ART allayed their exam anxiety.

**Table 1:** Student ratings of the usefulness of ART in classroom

**The ART…**	**Agree**	**Neutral**	**Disagree**
	**%**	**%**	**%**
…allowed me to identify my learning needs	95.9	0	4.2
…helped me maintain focus during lectures	93.1	5.6	1.4
…stimulated my interest in lectures	90.3	6.9	2.8
…allowed the lecturer to track our understanding	95.8	4.2	0
…motivated me to revise prior to the next session	66.7	20.8	11.1
…promoted my understanding of key concepts	87.3	11.3	1.4
…gave me an idea of exam style questions	97.2	2.8	0
…allayed my anxieties about the pharmacology exam	43.1	31.9	20.8

The percentage of student agreement with statements related to the usefulness of ART in the classroom across three categories: agree, neutral and disagree.

**Figure 2:**
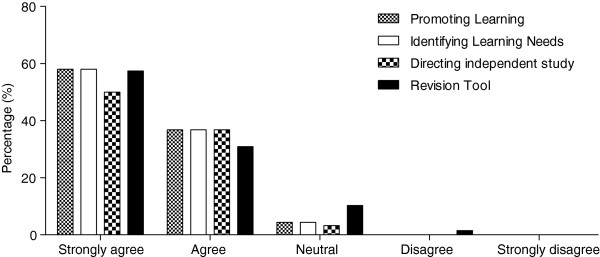
**Student ratings of the usefulness of ART feedback.** This graph presents students’ level of agreement with statements related to the usefulness of the individualised ART feedback in the following areas: promoting learning, identifying learning needs, directing independent study and as a revision tool.

Students’ levels of agreement with statements about the perceived usefulness of the individualised feedback are presented in Figure 2. When participant ratings of ‘strongly agree’ and ‘agree’ were combined, the majority of students agreed that the individual feedback helped promote their learning generally (96.4%); helped them to identify learning needs (95.6%); and helped them to direct their independent study (86.8%). Similarly, the majority of students (88.3%) agreed that the individualised feedback was a useful revision tool.

### Interviews

The thematic analysis of the interview data will be presented under a number of main themes. The first two themes “improved classroom atmosphere” and “improved in-class learning” relates to how students felt the individualised ART system influenced their experience of learning in the classroom. Under the theme “anxiety about performance”, students’ anxiety about using the individualised ART system is discussed. This anxiety related to both comparing their own scores to others in the classroom and to feeling nervous about knowing their own results and revealing their knowledge level to lecturers. Under the theme “enhanced self-management of learning”, the multiple ways in which students felt that the availability of individual formative feedback helped them to manage their learning are presented.

In the quoted excerpts, interviewees are labelled “A-G” respectively, and the interviewer is labelled “I”. The removal of an encouraging prompt from the interviewer has been indicated by the use of “…”, and “...” is used to denote the deletion of a section of text for the purpose of brevity.

#### Improved classroom atmosphere

All students felt that the use of ART enhanced the classroom experience in some way. Many participants felt that the use of ART added a fun and enjoyable aspect to lectures that otherwise contained a lot of difficult content:

"F: I think it sort of lightened the mood as well sometimes when it was really heavy."

Two participants suggested that the use of ART encouraged the student group to bond and become a closer unit by giving the class a common talking point:

"B: It did, you know, bring the group closer together... everyone’s in the same situation and it gives you all something you can talk about, a common interest."

As well as increased interaction between students, many participants felt that the use of ART encouraged a higher level of interactivity between students and lecturers, thereby allowing the lecturer to better gauge students’ progress on particular topics:

"C: I think it’s good for the lecturers as well…because then they can know whether they are teaching it in a way that people can understand or whether people are just going ”ok” and running off and saying I don't understand rather than having the courage to actually say “I don't understand it”."

#### Improved in-class learning

A number of positive learning outcomes in relation to the use of ART in the classroom were discussed. All participants felt that the use of ART was a helpful way of checking on their progress with the materials being taught:

"A: It gives you an idea at the end of the session if there’s some questions, if you have actually understood what you’ve been told."

As well as helping students to understand their progress, most participants discussed how the use of this system increased their motivation to focus in the lecture:

"B: I think that erm, having the keepads there, keeps you focused because you know you’re going to get asked on them like you’re constantly listening for key points and things so it just keeps you focused."

A further benefit of the use of ART in the classroom was that students valued the opportunity to practice exam-style questions before the real exam:

"B: I think to get used to the style of questions you would be asked they’re really helpful. Because you can parrot-fashion learn things but when you see them in an exam question that twists it slightly…then you panic."

Some students felt that the use of ART to test their knowledge helped them to consolidate any new information while they were in the lecture:

"G: They are a good way of stopping for a minute when you have learned all this stuff all these new concepts it was good to go over it, have a little breather and then carry on again."

Finally, in terms of positive learning outcomes of the use of ART in the classroom, some students discussed how getting the right answer to a question often promoted their confidence in the subject:

"D: If you got an answer right you thought yes I’ve got it sussed, I’m on the right track and it boosted your confidence."

In addition to confidence being promoted when students’ answered a question correctly, participants also discussed how the use of ART also reassured them when they got a question wrong as they could see they were not the only person struggling with the concepts:

"F: I think probably it was quite evenly spread in terms of knowledge and so it gave you the confidence to carry on because you realise actually you are not the only one that didn’t know that."

#### Anxiety about performance

Negative aspects of ART were also apparent in the interview data. Some participants reported feeling deflated when they got an answer wrong or when they perceived they were in the minority group of students who did not answer a question correctly:

"F: If the majority were right and I was one of the 10% that got it wrong that was horrible, that’s not so good."

Some participants also expressed initial anxiety about testing their knowledge using ART as they were worried about revealing their own level of pharmacology knowledge both to themselves and to the lecturer. Participant F was more worried about becoming more aware of her own lack of knowledge:

"I: And were the nerves related to using the keepads or was there something?"

"F: It was more highlighting the fact that I am stupid that was what I was more nervous about really, just you know, highlighting how little knowledge I have, or had, hopefully."

"I: Highlighting to yourself or others?"

"F: Both really and probably more concerning for myself on a personal level because you know realising your failings or your lack of knowledge or whatever is never a pleasant thing."

Other participants were very concerned about the lecturer’s perception of their knowledge level and this caused them some anxiety. Participant D discusses her initial fears about the tracking of student scores:

"D: I was terrified! I thought this is real big brother and we are going to be watched! So yeah, it increased my anxiety because I thought ‘yeah they are going to see how thick I actually am!’"

For several students, however, their anxiety about answering questions incorrectly lessened over time, as they focused on what they learned from getting a question incorrect:

"A: At first it was a bit like, ohhh, and you beat yourself up about getting the wrong answer but you know, by sort of week two week three you sort of think you know, right...it’s just a learning thing then, rather than feeling “oh God, I got it wrong” or “oh, I got it right”."

For two participants, the confidentiality associated with the use of individualised ART allayed some of their initial anxieties about the system, as they realised that only themselves and the lecturer would have access to their score:

"G: First of all I was a bit apprehensive because I thought oh does everyone else in the room know your results but then I thought the fact that they didn’t, I thought was great."

#### Enhanced self-management of learning

Interviewees discussed how the receipt of individualised feedback, in the form of an e-mail of their ART results, enhanced their self-management of their learning. All participants described the benefits of receiving an objective reminder of their ART scores so they could keep track of their own progress:

"F: The email feedback really drew it all together and reminded you ok we did this in the class, we had fun but actually here is the bits that you did well and didn’t do so well and I think that was nicely done."

Students felt that the e-mail feedback helped them to identify their learning needs and helped some students direct their revision:

"D: “I was like well these are the areas I need to revise or go back over.”"

While participants found the feedback useful in terms of targeting their learning needs, they did not necessarily change their revision strategy entirely, as most tended to revise all topics regardless of their feedback scores:

"A: I thought well perhaps I need to do more on that subject but then I went through the whole thing anyway you know."

Some students used the feedback sheets as self-assessment tools in preparation for their exams. For example, participant C asked her husband to test her on the questions using the ART feedback sheet:

"C: I got my husband to read them out and see which ones I got right and the ones I didn't get right I went away and looked at and saw what I got wrong and used it that way."

The individualised feedback sheets helped two students decide that they needed to seek extra lecturer support for certain topics:

"D: If the same question came up and I was still getting them wrong I obviously realised that I was either not reading the question right……or else I just had a mental block and I just needed to ask somebody."

When students were asked whether they thought lecturers should contact them to follow-up on their feedback scores to offer assistance, most students felt that this was unnecessary and preferred the current system where they had control over the implications of their individualised feedback scores. Some students felt that if the lecturer did follow-up on their scores and contact them this would cause them anxiety:

"F: I would probably be irritated by that actually and it probably wouldn’t do my confidence any good whatsoever……yes it think for me I would just go into panic mode."

Most students were content with the level of personal control they had to use the individualised feedback in the way they chose to:

"F: At the end of the day we are adult learners so you know you’ve got a problem you can go out and source to find support, you can’t expect to be spoon-fed everything."

### Exam score correlation

There was a strong positive significant correlation between the mean individual ART score of each student (n=107) and their result in the summative pharmacology examination (Spearman’s rho = 0.71, p < 0.01).

## Discussion

Our previous work has demonstrated that students enjoy using the ART and feel that it helps promote understanding and integration of pharmacological understanding and independent learning [8]. The aims of the current study were to explore student perceptions of the use of personal ART handsets and the associated individualised feedback and to determine whether ART performance (as measured by feedback scores) was associated with summative exam outcomes.

Key themes which emerged from the student questionnaires and interviews, overlapped somewhat with those reported in our previous study of anonymous ART use, including identification of learning needs, increased motivation and consolidation of knowledge [8]. Whilst this may not seem surprising, it is important to note that the individual ‘tracked’ nature of the use of the ART did not adversely affect students’ positive perceptions of using ART. Whilst students did initially exhibit some anxiety with regard to the individualised use of the ART, as was previously suggested in the literature [3], some of this anxiety was related to a lack of understanding of the nature of the system. Student’s anxiety seemed to have been allayed as they got used to using the system, received feedback, and fully understood the level of anonymity inherent in the system.

Themes novel to student use of personal ART identified include: the ability of the students and lecturers to “track” learning at a deeper level, the ability for students to keep track of own performance (correct and incorrect answers), and target their own learning needs effectively. Additionally, we identified a significant correlation between formative performance via individualised feedback and summative exam scores. Although we are unable to attribute the correlation between feedback and summative exam scores, this finding provides confidence in our ability to identify students at an early stage who may require additional support.

Students valued the opportunity to track their learning through the personalised ART formative feedback. In addition, the feedback received by students was completely objective which served as an accurate reminder for students of how they performed on each of the topic areas in the pharmacology lectures. As described previously, early formative feedback is particularly important in developing confidence in this cohort of students, with 87% expressing poor or moderate biology knowledge at the start of the course. In our previous study of the use of anonymous ART , students typically noted down only those questions which they answered incorrectly to review later. This could potentially skew the students’ perception of personal performance; the personalised feedback received by students in the present study outlined both incorrect and correct answers.

While objective formative feedback has been suggested to be key to improving student performance [19,20], other literature has stressed the importance of detailed individual feedback [21]. The ART system utilised in the present study allows for the provision of feedback which meets all these requirements, as it provides frequent, detailed, individual and objective feedback and as such should act to improve student confidence [22] and knowledge [23]. The availability of formative feedback from the first week of the course allowed students to develop confidence in their abilities and thus enabled them to target areas of weakness more effectively.

Despite a strong, positive correlation between formative performance (feedback scores) and corresponding summative exam marks, only 43% of students agreed that the use of ART allayed exam anxieties. This is likely the result of the fact that many students on this course are anxious about their exam results as they may be expected to achieve the NMP qualification to maintain their current job role. Whilst students did not want tutors to contact them expressing concerns regarding their level of understanding, knowledge of the strong correlation between formative feedback performance and exam performance may further encourage these students to take responsibility for their own learning and seek appropriate help and support.

Further research in the area of individualised feedback using ART technology could seek to clarify whether use of individualised feedback is related to better exam performance. This was not achievable in the current study due to absence of a control group to compare students who had access to feedback and those who do not. As NMP students had previously reported benefits of using the anonymous ART system [8], the authors felt that it would be unethical to give ART access to only a proportion of the students on the course purely for the purposes of comparing exam results. However, such an experimental study could add weight to the current evidence of the potential benefits of using this individualised feedback system technology in the classroom.

## Conclusions

The current study demonstrates for the first time that the use of individualised ART coupled with regular personalised formative feedback acts to enhance student confidence and promote learning over and above that seen with anonymous ART system, with the benefits outweighing the initial anxiety the system provokes in students. The individual feedback also correlates well with summative exam scores and acts as a useful reference for both staff and students in terms of exam readiness. ART could be utilised in this way to enhance learning and understanding of a variety of subjects across a range of different students.

## Competing interests

The authors declared that they have no competing interests.

## Authors' contributions

AM and JSL conceived of, designed the study and obtained the funding; AM, OM and JSL conducted the study and analysed the data. AM, OM and JSL drafted the manuscript. All authors have read and approved the final manuscript.

## Pre-publication history

The pre-publication history for this paper can be accessed here:

http://www.biomedcentral.com/1472-6920/12/113/prepub

## References

[B1] Medina MS Medina PJ Wanzer DS Wilson JE Er N Britton ML Use of an audience response system (PRS) in a dual-campus classroom Am J Pharm Educ 2008 72 2 38 10.5688/aj720238 18483604 PMC2384213

[B2] Cain J Black EP Rohr J An audience response system strategy to improve student motivation, attention and feedback Am J Pharm Educ 2009 73 2 21 10.5688/aj730221 19513159 PMC2690899

[B3] Nayak L Erinjeri JP Audience response systems in medical student education benefit learners and presenters Acad Radiol 2008 15 3 383 389 10.1016/j.acra.2007.09.021 18348839

[B4] Alexander CJ Crescini WM Juskewitch JE Lachman N Pawlina W Assessing the integration of audience response system technology in teaching of anatomical sciences Anat Sci Educ 2009 2 4 160 166 10.1002/ase.99 19670428 PMC2802184

[B5] Doucet M Vrins A Harvey D Effect of using an audience response system on learning environment, motivation and long-term retention, during case-discussions in a large group of undergraduate veterinary clinical pharmacology students Med Teach 2009 31 12 e570 e579 10.3109/01421590903193539 19995158

[B6] Ernst H Colthorpe K The efficacy of interactive lecturing for students with diverse science backgrounds Adv Physiol Educ 2007 31 41 44 10.1152/advan.00107.2006 17327581

[B7] DeBourgh GA Use of classroom ‘clickers’ to promote acquisition of reasoning skills Nurs Educ Pract 2008 8 76 87 10.1016/j.nepr.2007.02.002 18291324

[B8] Lymn J Mostyn A Audience response technology: Engaging and empowering non-medical prescribing students in pharmacology learning BMC Med Educ 2010 10 1 73 10.1186/1472-6920-10-73 20979620 PMC2978227

[B9] Jones S Henderson D Sealover P "Clickers" in the classroom Teach Learn Nurs 2009 4 1 2 5 10.1016/j.teln.2008.06.001

[B10] Paschal CB Formative assessment in physiology teaching using a wireless classroom communication system Adv Physiol Educ 2002 26 4 299 308 12444002 10.1152/advan.00030.2002

[B11] Gauci SA Dantas AM Williams DA Kemm RE Promoting student-centered active learning in lecctures with a personal response system Adv Physiol Educ 2009 33 60 71 10.1152/advan.00109.2007 19261762

[B12] Rush BR Hafen M Jr Biller DS Davis EG Klimek JA Kukanich B The Effect of Differing Audience Response System Question Types on Student Attention in the Veterinary Medical Classroom J Vet Med Educ 2010 37 2 145 153 10.3138/jvme.37.2.145 20576903

[B13] Porter AG Tousman S Evaluating the Effect of Interactive Audience Response Systems on the Perceived Learning Experience of Nursing Students J Nurs Educ 2010 49 9 523 527 10.3928/01484834-20100524-10 20509583

[B14] Department of Health 2006 London: Medicines Matters

[B15] Manias E Bullock S The educational preparation of undergraduate nursing students in pharmacology: perceptions and experiences of lecturers and students Int J Nurs Stud 2002 39 757 769 10.1016/S0020-7489(02)00018-4 12231032

[B16] Morrison-Griffiths S Snowden MA Pirmohamed M Pre-registration pharmacology: is it adequate for the roles that nurses are expected to fulfil? Nurse Educ Today 2002 22 447 456 12387758

[B17] King RL Nurses perceptions of their pharmacology educational needs J Adv Nurs 2004 45 392 400 10.1046/j.1365-2648.2003.02922.x 14756833

[B18] Patterson B Kilpatrick J Woebkenberg E Evidence for teaching practice: The impact of clickers in a large classroom environment Nurse Educ Today 2010 30 7 603 607 10.1016/j.nedt.2009.12.008 20044180

[B19] Carrillo-de-la-Peña MT’ Baillès E Caseras X Martínez A Ortet G Pérez J Formative assessment and academic achievement in pre-graduate students of health sciences Adv Health Sci Education Theory Pract 2009 14 61 67 10.1007/s10459-007-9086-y 17972153

[B20] Norman G Neville A Blake JM Mueller B Assessment steers learning down the right road: Impact of progress testing on licensing examination performance Med Teach 2010 32 496 499 10.3109/0142159X.2010.486063 20515380

[B21] Rushton A Formative assessment: a key to deep learning? Med Teach 2005 27 6 509 513 10.1080/01421590500129159 16199357

[B22] Chur-Hansen A Koopowitz LF Formative feedback in teaching undergraduate psychiatry Acad Psychiatry 2005 29 66 68 10.1176/appi.ap.29.1.66 15772407

[B23] Velan GM Jones P McNeil HP Kumar RK Integrated online formative assessments in the biomedical sciences for medical students: benefits for learning BMC Med Educ 2008 8 52 10.1186/1472-6920-8-52 19032738 PMC2613879

